# Lipomyelomeningocele in a Newborn: A Case Report

**DOI:** 10.7759/cureus.87596

**Published:** 2025-07-09

**Authors:** Karla V Soria, Ignacio J Contreras, Andrew Kobets, Lourdes Cohen

**Affiliations:** 1 Pediatrics, Flushing Hospital Medical Center, Flushing, USA; 2 Pediatrics/Neonatal Division, Flushing Hospital Medical Center, Flushing, USA; 3 Neurosurgery, Children's Hospital at Montefiore, Bronx, USA

**Keywords:** congenital, congenital abnormalities, lipomyelomeningocele, newborn infant, spinal dysraphism, tethered spinal cord

## Abstract

Lipomyelomeningocele (LMMC) is an uncommon type of closed spinal defect that falls under the broader category of spine bifida. This congenital condition may present at birth as a lumbosacral mass or manifest later in life with neurologic symptoms. In this report, we describe a neonate who was diagnosed with LMMC at birth and underwent timely surgical intervention during the neonatal period. The procedure was successful and the patient experienced no adverse outcome. This case highlights the importance of early diagnosis and prompt surgical repair.

## Introduction

Lipomyelomeningocele (LMMC) is a type of closed neural tube defect, where neural elements are integrated into a spinal lipoma. This rare condition affects one to six individuals per 100,000 live births. It is a closed defect due to abnormal central nervous system development, involving primary or secondary neurulation. During primary neurulation, the neural tube forms and the ectoderm separates to create the skin of the spine. If disjunction occurs, mesoderm can enter the neural tube and create a lipoma that disrupts normal anatomy. Secondary neurulation is the formation of the spine below S2 through the cavitation and fusion of mesoderm with the primary neural tube. This process can be interrupted by mesoderm migration. Secondary neurulation is not completely understood, however, there are some theories suggesting the involvement of morphogenetic determinants, with potential key genes including Sonic Hedgehog and Pax family transcription factors [1]. In LMMC, the neural-lipoma junction is outside the spinal canal, extending dorsally through a bony defect. The lipoma exits the dura defect, passes through a fascial defect, and connects with subcutaneous tissue, tethering the spinal cord and making it vulnerable to stretch injuries during growth or repetitive movements [1].

LMMCs are classified into four types, as described by Chapman, depending on the location of the neural-lipoma interface: (1) dorsal, if the interface is at the dorsum of the spinal cord and the conus medullaris is spared; (2) caudal, if the filum terminal or the conus medullaris is involved; (3) transitional, when dorsal and caudal components are involved with nerve roots crossing the lipoma; and (4) where the spinal cord exits the spinal canal distally within the fatty mass [2].

## Case presentation

A 26-year-old Hispanic woman, gravida 2, para 0, presented at 40 weeks gestation for an elective induction of labor. Due to prolonged labor course, a low transverse cesarean section was performed. The pregnancy was complicated by the presence of uterine fibroids. A first-trimester prenatal ultrasound was normal; however, during the second trimester, fetal sac views were suboptimal, raising concerns about a possible spinal abnormality.

A female infant weighing 3075 grams was delivered by cesarean section. Apgar scores were 9 at both 1 and 5 minutes. The placenta was intact.

**Figure 1:**
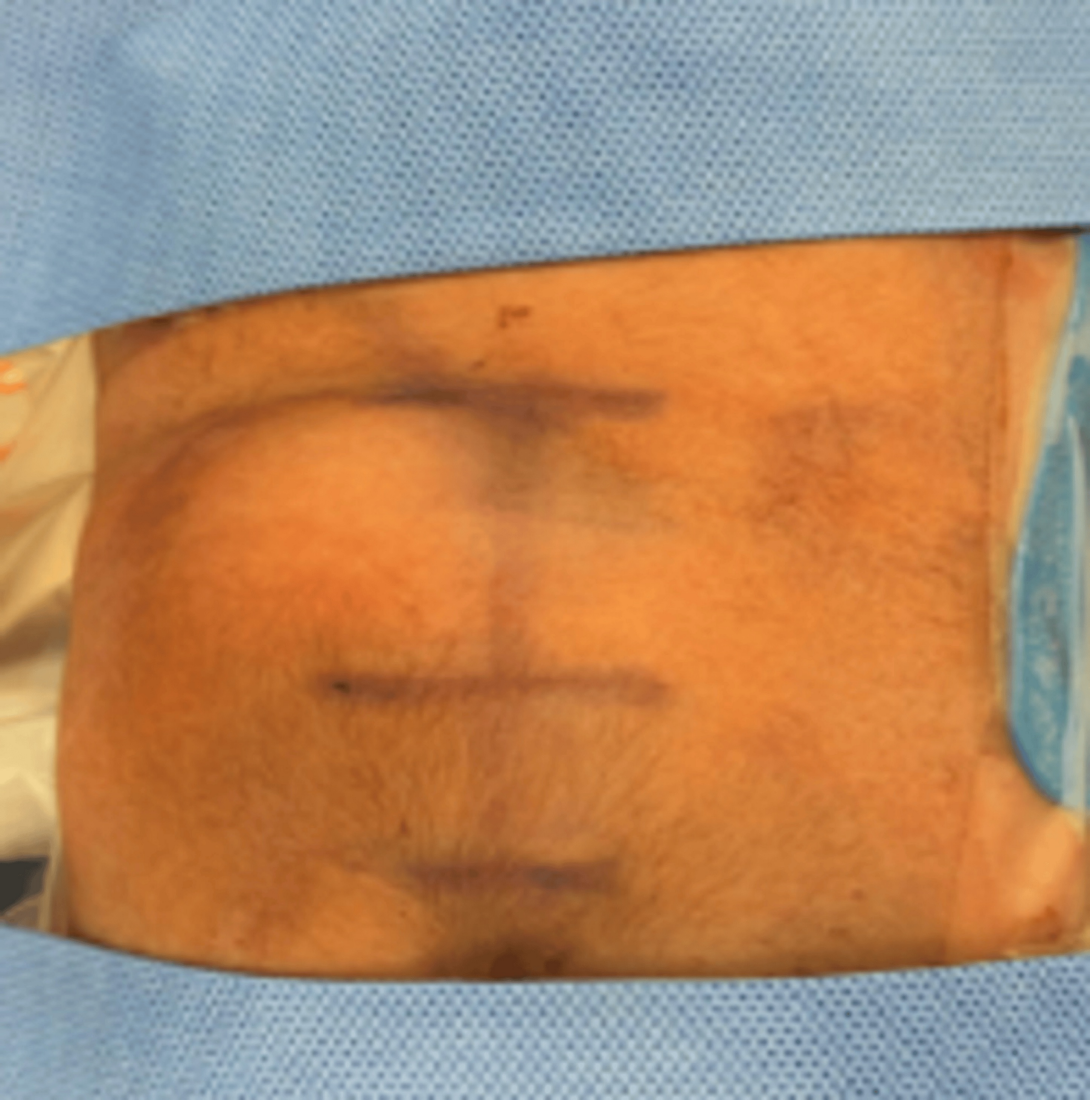
A 4x3.5 cm circular, soft, nontender, nonpulsatile mass to right of lumbosacral region

On admission physical exam, a closed sacral dimple with a left palpable soft mass in the lumbosacral region was noted (Figure 1). Left hip examination was positive for a click. Complete neurological examination was within normal limits, including deep tendon reflexes and sensation.

**Figure 2:**
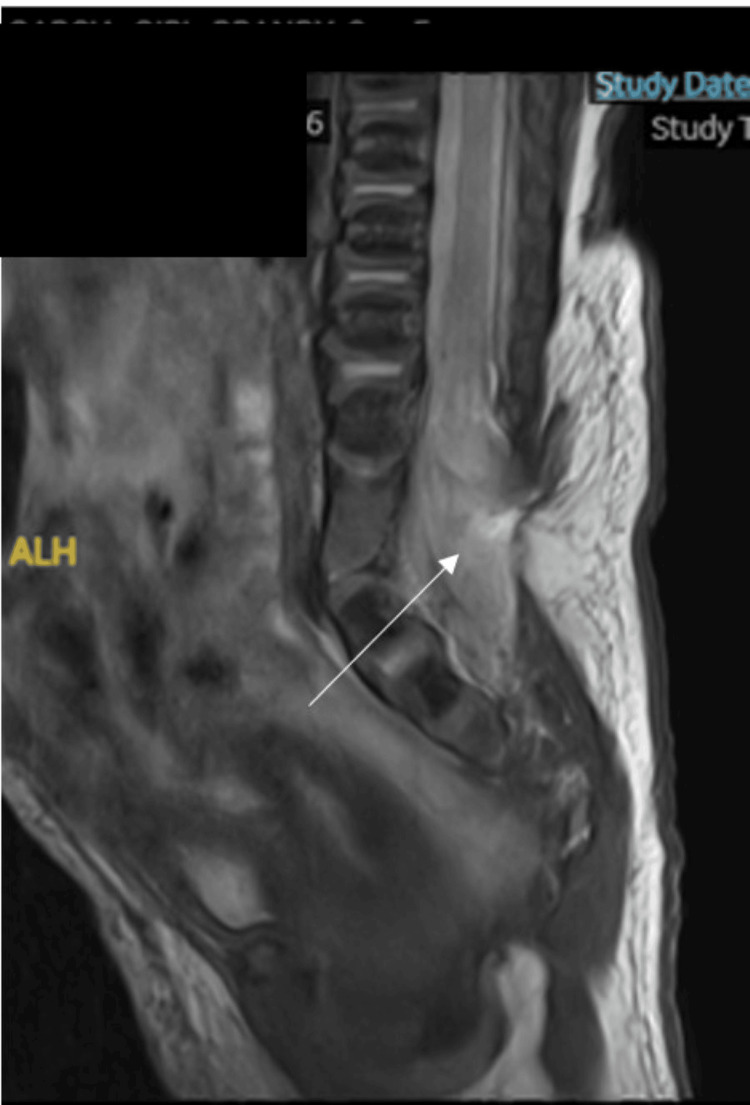
Spine MRI

Ultrasound confirmed left hip dysplasia and suspected spinal dysraphism. Brain MRI findings were within normal limits. MRI of the spine demonstrated a low-lying conus medullaris and a large posterior bony defect in the lower lumbar region, through which the spinal cord was herniated. The distal spinal cord was tethered to the posterior spinal elements. No evidence of syringomyelia or cerebellar tonsillar descent was observed, supporting the diagnosis of LMMC (Figure 2).

**Figure 3:**
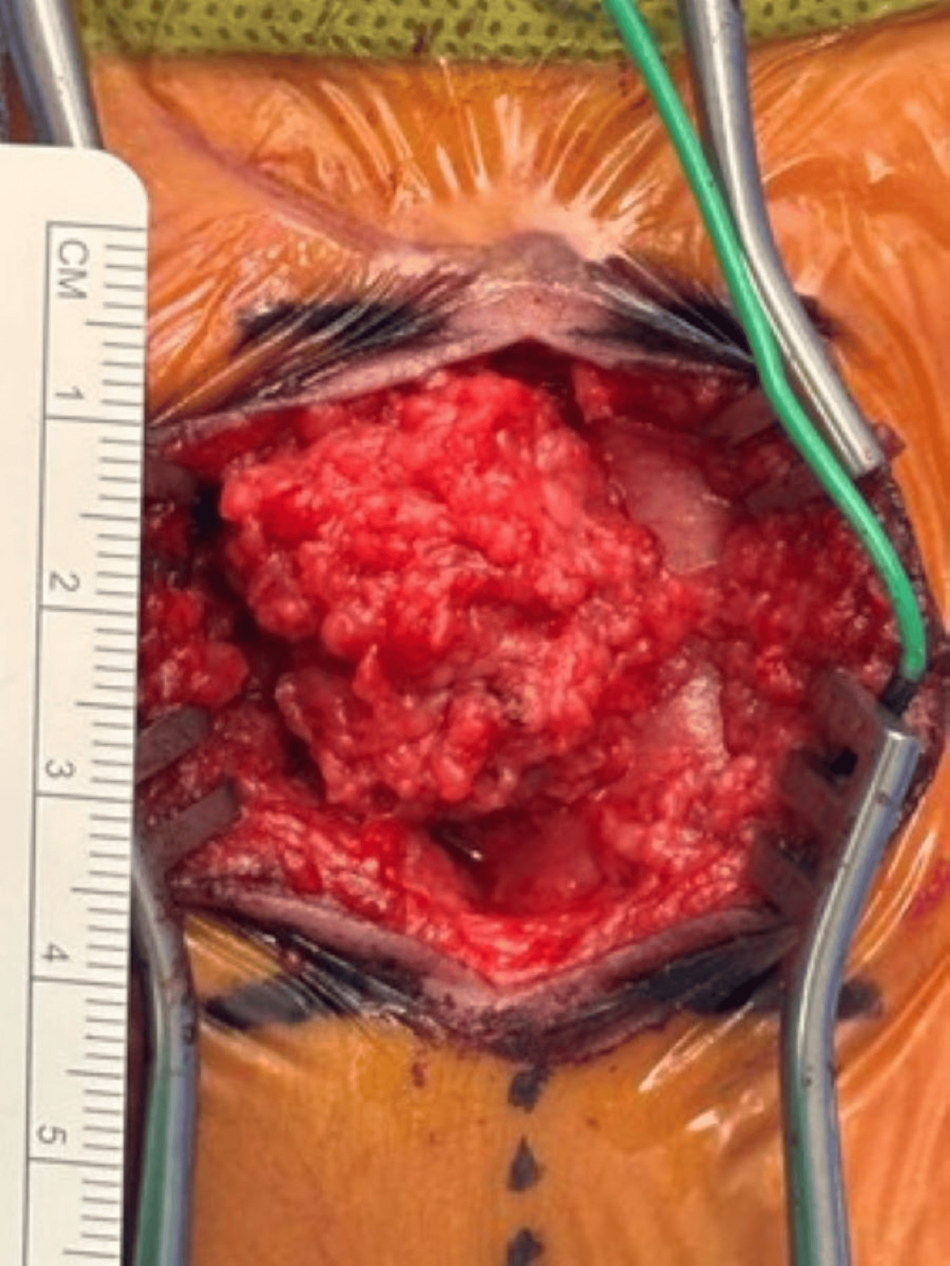
Lipomatous tissue encasing the neural placode

She was transferred at six days of life (DOL) to a higher level of care hospital for surgical repair. The procedure was performed with no complications. A large lipomatous mass enveloped in the neural placode of the spinal cord was found intraoperative (Figure 3).

**Figure 4:**
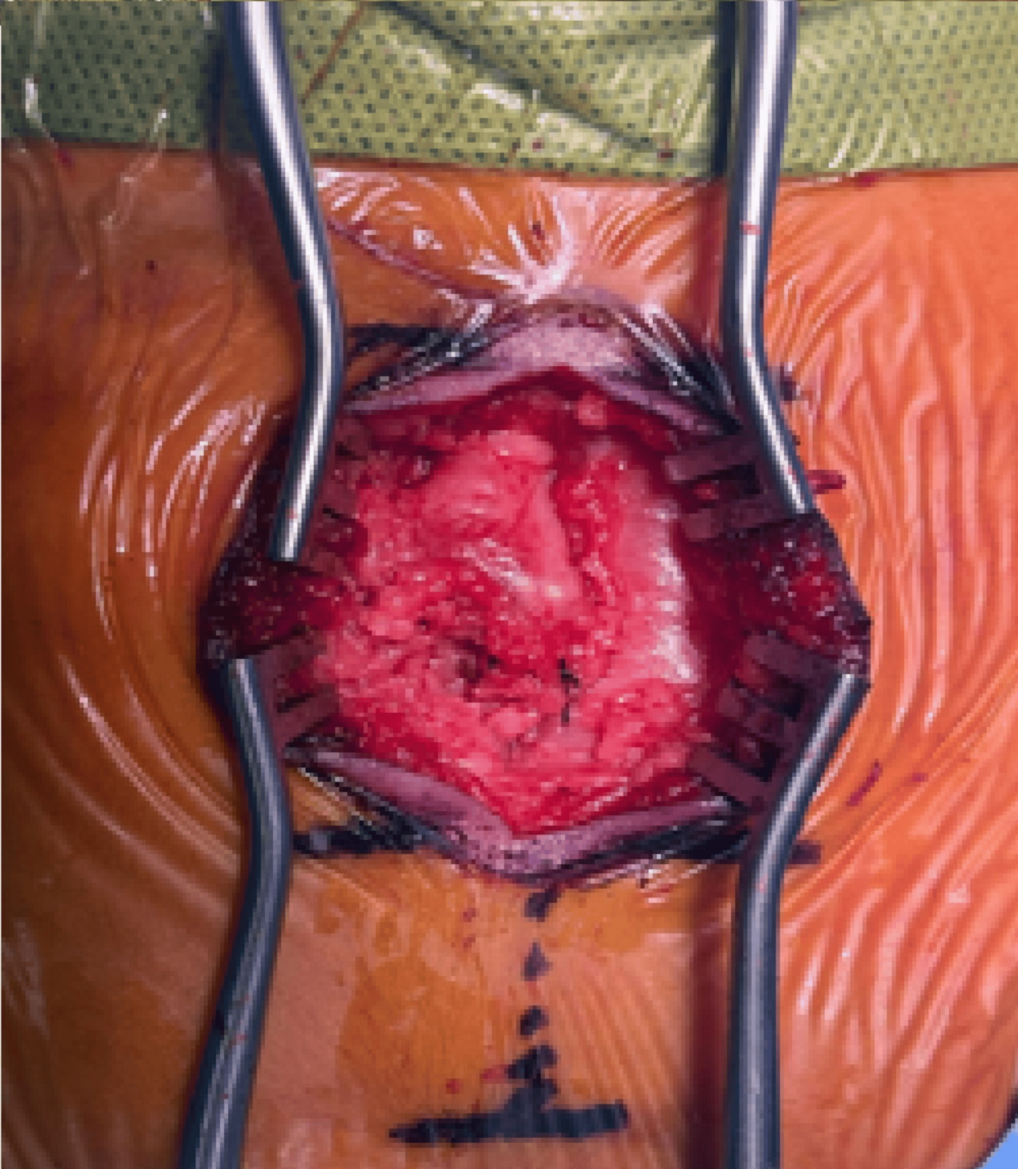
Spinal cord dissected from the lipoma

The cord was carefully dissected from the lipoma, allowing it to bring the dura and cord back into the spinal canal and to a normal anatomic position (Figure 4).

**Figure 5:**
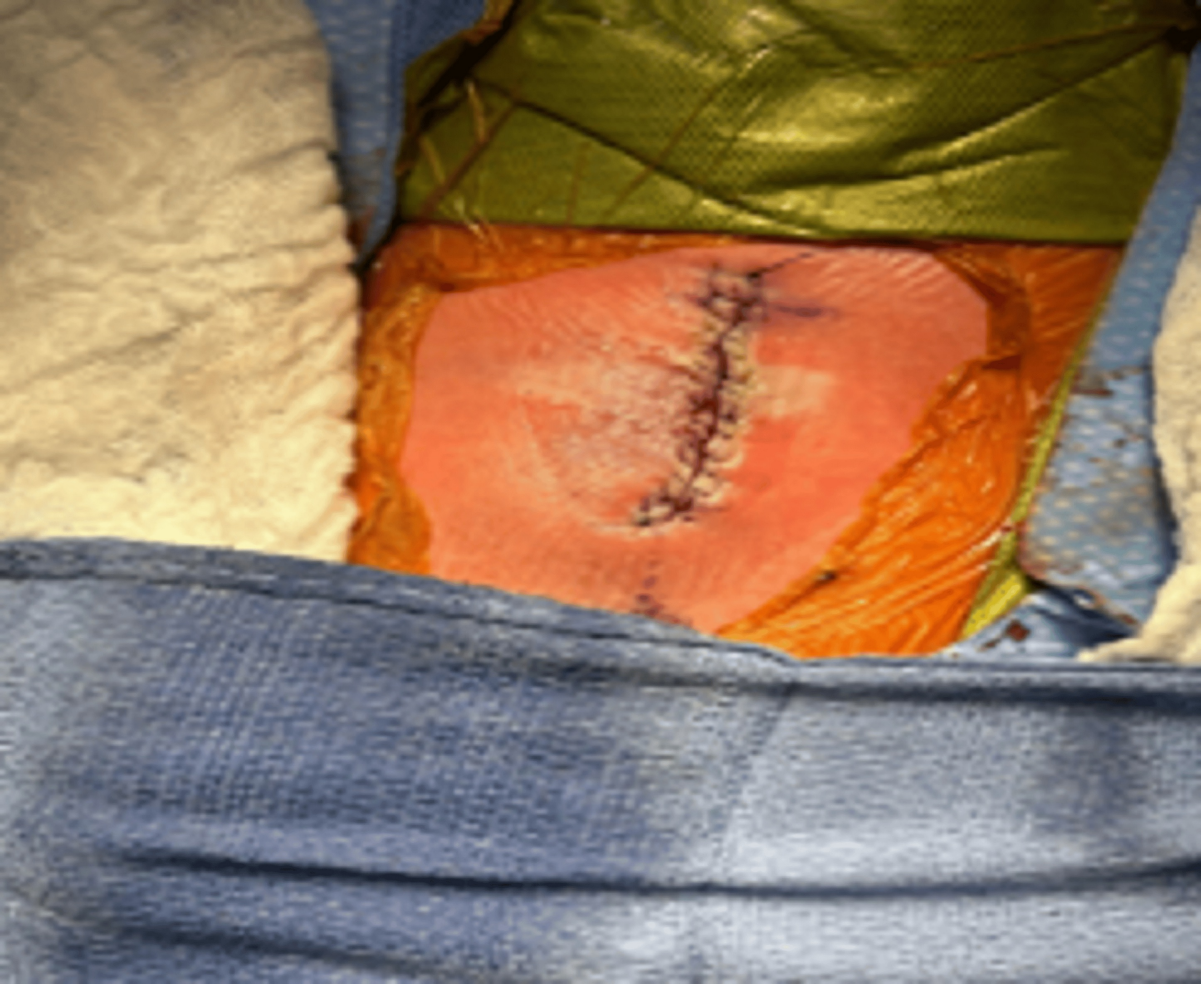
LMMC repaired and closed LMMC: lipomyelomeningocele

The closure of the fat layers and sealing of the dura was performed, followed by an additional resection of the remaining fat to achieve a cosmetically appealing closure (Figure 5).

She was discharged, and subsequent follow-up visits revealed normal neurologic examinations, with no evidence of motor or sensory compromise.

## Discussion

Our case report identified a neonate who presented with a paraspinal mass at the time of birth associated with left hip dysplasia. MRI confirmed the diagnosis of LMMC.

This case is in accordance with the epidemiology of LMMC and other forms of closed spinal dysraphism which indicate higher frequency among individuals of Hispanic ethnicity compared to non-Hispanic White populations. This characteristic suggests a potential ethnic, genetic, environmental, or socioeconomic association that is not fully understood and warrants further research [3].

Our case was not associated with Chiari malformation which can occur in 13%, spinal dermal sinuses, anal stenosis (1%), or Down syndrome (1%). We did not find any visible capillary malformation, infantile hemangioma, or any other caudal malformations of organ systems like, cloacal exstrophy, VACTERL (vertebral defects, anal atresia, cardiac defects, trachea-esophageal fistula, renal anomalies, and limb abnormalities) [4,5].

The patient had a successful surgical repair on day 10 of life. The surgical management of LMMCs remains controversial, especially in patients with asymptomatic diseases. Various studies have suggested risks of progressive neurologic, urologic, and orthopedic deficits in patients with LMMCs if untreated. Other studies have shown progressive neurologic and urologic deficits in patients who were operated on prophylactically as well [6,7]. These variable outcomes may be due to the different symptoms that were assessed, the duration of follow-up, the type of malformation, and the timing of surgery [8].

Factors that may affect the treatment outcome of LMMCs include age, gender, morphology, the presence and severity of neurological symptoms, and the absence and presence of an associated spinal cord syrinx, of which the morphology is considered the most crucial factor affecting outcome [1]. One study suggests that those with symptomatic LMMCs had improvement or preservation of their pre-operative neurological function at follow-up visits, especially if corrected soon after the development of the deficit [9]. Some neurosurgeons suggest that early surgical intervention can be performed prophylactically to prevent neurological sequelae secondary to a tethered cord [10].

## Conclusions

This case report describes the successful neonatal surgical management of an asymptomatic infant with LMMC, without evidence of spinal cord syrinx. The favorable outcome contributes to the growing body of literature on early intervention strategies for neonates with LMMC.
